# Can Expanded Bacteriochlorins Act as Photosensitizers in Photodynamic Therapy? Good News from Density Functional Theory Computations

**DOI:** 10.3390/molecules21030288

**Published:** 2016-02-29

**Authors:** Gloria Mazzone, Marta E. Alberto, Bruna C. De Simone, Tiziana Marino, Nino Russo

**Affiliations:** 1 Dipartimento di Chimica e Tecnologie Chimiche, Università della Calabria, I-87036 Arcavacata di Rende, Italy; gloria.mazzone@unical.it (G.M.); bruna.desimone@unical.it (B.C.D.S.); tiziana.marino65@unical.it (T.M.); 2 Chimie ParisTech, CNRS, Institut de Recherche de Chimie Paris (IRCP), PSL Research University, F-75005 Paris, France; marta.alberto@chimie-paristech.fr

**Keywords:** DFT, TD-DFT, electronic spectra, spin-orbit coupling constants, bacteriochlorins

## Abstract

The main photophysical properties of a series of expanded bacteriochlorins, recently synthetized, have been investigated by means of DFT and TD-DFT methods. Absorption spectra computed with different exchange-correlation functionals, B3LYP, M06 and ωB97XD, have been compared with the experimental ones. In good agreement, all the considered systems show a maximum absorption wavelength that falls in the therapeutic window (600–800 nm). The obtained singlet-triplet energy gaps are large enough to ensure the production of cytotoxic singlet molecular oxygen. The computed spin-orbit matrix elements suggest a good probability of intersystem spin-crossing between singlet and triplet excited states, since they result to be higher than those computed for 5,10,15,20-tetrakis-(m-hydroxyphenyl)chlorin (Foscan©) already used in the photodynamic therapy (PDT) protocol. Because of the investigated properties, these expanded bacteriochlorins can be proposed as PDT agents.

## 1. Introduction

Photodynamic therapy (PDT) is a minimally invasive therapeutic intervention currently used for the treatment of a variety of cancers and non-oncological disorders [1,2,3]. The death of diseased cells is achieved by the use of visible or near-infrared radiation to activate a light-absorbing compound (photosensitizer, PS), which, in the presence of molecular oxygen, entails the formation of reactive oxygen species responsible of apoptosis, autophagy, or necrosis of the treated cells. The PDT procedure follows essentially three steps: (i) the irradiation in the range of 600–800 nm, where tissues are more permeable to light, induces the excitation of the PS from its ground state (S_0_) to the first excited one (S_1_); (ii) the S_1_ state undergoes efficient intersystem crossing that generates the first excited triplet state of the molecule, T_1_; (iii) T_1_ state can then relax back to the ground state following two types of processes: type I and type II photoreactions. In the former case, the PS in the T_1_ state abstracts an electron from a reducing molecule in its vicinity, giving rise to highly reactive species (*i.e.*, O_2_^−^, NO, ROO, RO) able to damage the targeted cells. In the latter one, supposed as the predominant process, the energy of the T_1_ state is transferred to the molecular oxygen (^3^Σ_g_ˉ) to yield singlet oxygen ^1^O_2_ (^1^Δ_g_), which represents the putative cytotoxic agent. Accordingly, together with specific chemical properties, an efficient PDT photosensitizer should possess: (i) a maximum absorption in the so-called therapeutic window (600−800 nm), allowing the treatment of deeper tumors; (ii) a high intersystem spin-crossing probability; (iii) a singlet-triplet energy gap greater than 0.98 eV (the amount of energy required to activate the molecular oxygen) and, consequently, good singlet oxygen quantum yield (Φ_Δ_).

In the last decades, several porphyrin-like systems and their metal complexes have been extensively studied at both theoretical [4,5,6,7,8,9,10,11,12] and experimental [13,14] levels in view of their potential application in photodynamic therapy. These compounds present low dark toxicity, thermodynamic stability and interesting absorption properties in the Q region of the spectrum, which can be further modulated by varying the π delocalization. Moreover, they can easily form metal complexes and can be successfully functionalized with heavy atoms with a consequent increasing of the intersystem spin crossing efficiency [15]. Several porphyrin-like compounds and their complexes are already used in PDT and some of them are currently in advanced phases of clinical trials [16].

Among porphyrin-like systems, bacteriochlorins have emerged as a class of compounds that meet most of the requirements for ideal PDT agents [17,18,19,20,21,22,23,24,25,26,27,28,29,30,31,32,33]. They are tetrapyrrole compounds with two opposing pyrroline rings, resulting from the reduction of the two pyrrole rings in the tetrapyrrole macrocycle of the correspondent porphyrins. The macrocycle structure occurs naturally in photosynthetic pigments (bacteriochlorophylls a and b) found in purple photosynthetic bacteria [34]. The bacteriochlorins are characterized by very high molar absorption coefficients in the therapeutic window (600–800 nm) and, accordingly, may be effective at lower concentrations. Therefore, the presence of pyrroline moiety has a noticeable effect on the absorption spectra, as neither chlorins nor porphyrins absorb in the NIR spectral region that ensures a deeper penetration of light in tissue compared to porphyrin derivatives.

Recent advances in this field have shown how new synthetic bacteriochlorins attained the photostability, long-lived triplet states, and high quantum yields in the generation of ROS, all of these being essential properties for PDT photosensitizers [35].

**Scheme 1:**
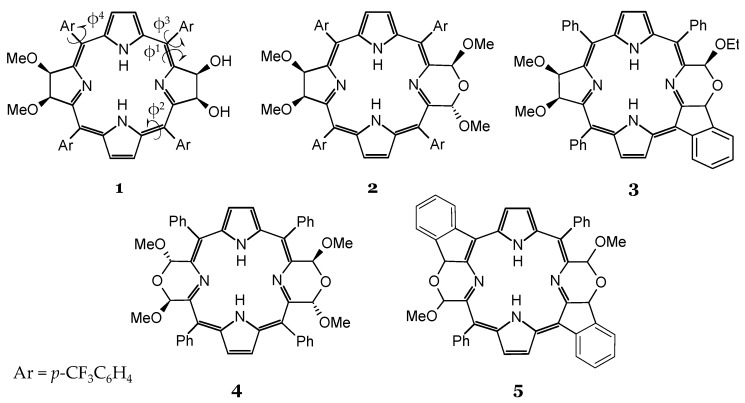
Chemical structures of the investigated bacteriochlorins **1**–**5**.

Herein, the photophysical properties of a series of expanded bacteriochlorins (see Scheme 1) have been investigated with the aim to assess whether some of these recently synthesized compounds [36] could be proposed as photosensitizers in PDT. As largely documented several photophysical properties can be accurately predicted and rationalized from first principles calculations [4,5,6,7,8,9,10,11,12,37,38,39,40,41,42,43,44,45]. Among these, the maximum absorption wavelengths, the singlet-triplet energy gaps and key information on the intersystem spin crossing efficiency are the most important ones for PDT application. The present study provides a screening of the expanded bacteriochlorins properties which can help to select the best candidate as PDT agent.

## 2. Results and Discussion

### 2.1. Ground State Properties

**Figure 1:**
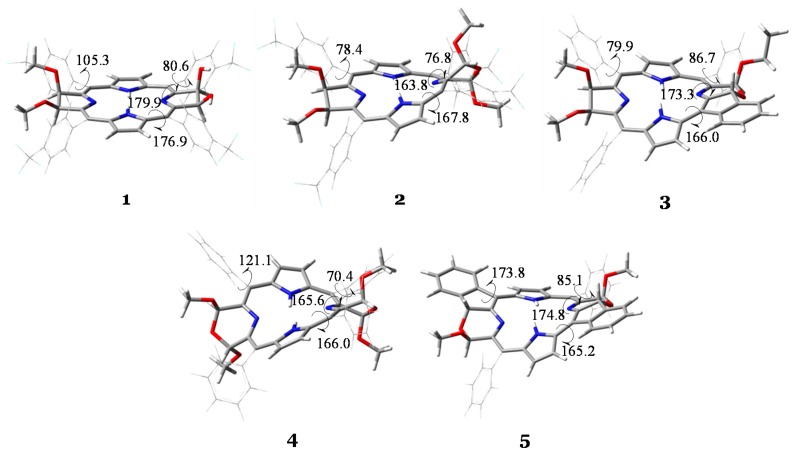
Optimized structures of compounds **1**–**5** computed at B3LYP/6-31G* level of theory. Torsion angles, φ^1^, φ^2^, φ^3^ and φ^4^ are reported in degrees.

All the investigated bacteriochlorins **1**–**5** have been optimized without constrains at the B3LYP/6-31G* level of theory and the resulted structures are reported in Figure 1 together with some key structural parameters (see also Scheme 1).

The structural expansion of bacteriochlorins made by Samankumara *et al.* [36] to enhance the photophysical properties includes (i) the introduction of the morpholino group in the pyrroline ring (compounds **2** and **4**) and (ii) the fusion of the *meso*-phenyl group to the morfoline moiety forming a five-membered ring by a direct β-to-*o*-phenyl linkage (compounds **3** and **5**).

Starting from the most planar structure (**1**), the inclusion of the morpholino group, compound **2**, entails a distortion of the entire macrocycle because of the strain resulted by the insertion of the oxygen atom between the two sp^3^-hybridized pyrrolidone β carbons. This effect can be observed also in compound **4**, which differs from **2** for the inclusion of a further morpholino moiety and the substitution of the *meso*-Ar groups with the Ph ones. Indeed, the φ^1^ torsion angle, which is 179.9 degrees in **1**, becomes 163.8 and 165.6 degrees in **2** and **4**, respectively. The fusion of one or two *meso*-phenyl group to the morfoline ring(s) in **3** and **5**, respectively, produces a less noticeable effect on φ^1^ parameter. In fact, it turns out to be very close to the planarity value (being 173.3 and 174.8 degrees for **3** and **5**, respectively), due to the extended π conjugation of the macrocycle that includes the almost coplanar *meso*-Ph groups. However, the latter compounds are not completely planar as the effect of the insertion of the oxygen atom in the six-membered ring is still quite pronounced. Indeed, looking at the φ^2^ torsion angles, it is possible to observe that, while in **1** it is found to be 176.9 degrees, in the other molecules it ranges from 165.2 (**5**) to 167.8 (**2**) degrees, representative of a nonplanar structures for **3** and **5** molecules, as well as for **2** and **4**. In all cases, the orientation of the *meso*-substituents (Ar or Ph), described by the φ^3^ and φ^4^ torsion angles defined above, is approximately orthogonal to the porphyrin-like plane (70.4 < φ^3^ < 86.7 and 79.9 < φ^4^ < 121.1 degrees), with the exception of compound **5**, for which the establishment of the β-to-*o*-phenyl linkage makes the latter parameter obviously close to planarity (φ^4^ = 173.8).

From the structural analysis, it is clear that the twist of both the morpholino groups in compound **4** introduces the largest degree of strain with respect to the other compounds, making **4** the most distorted molecule. Molecule **5** results to be most rigid one because the presence of the fused *meso*-groups to the morpholino ones entails an extension of the π conjugation.

Although our discussion is based on the isolated molecules, the interaction with DNA could affect the torsion angles and, consequently, modify their photophysical properties [46]. Since the porphyrin-like systems are not well selective, ^1^O_2_ does not induce oxidative stress only on DNA but also on other cellular components, such as membranes and proteins.

**Figure 2:**
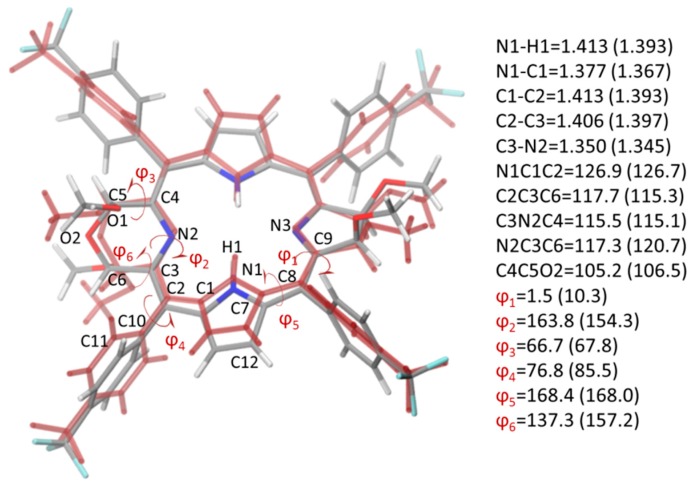
Superimposition of the optimized B3LYP/6-31G* and crystallographic (in red) structures of molecule **2**. Selected computed and crystallographic (in parenthesis) geometrical data, bond distances in Å and valence and torsion angles in degrees, are reported.

A comparison between experimental and computed structural parameters for molecule **2,** reveals a satisfactory agreement. A RMSD (root mean square deviation) value equal to 0.966 Å has been obtained considering all the geometric parameters. A superimposition of the two structures and key structural data are reported in Figure 2.

The difference between the two structures is mainly due to the distortion degree of the four N-rings with respect to the ideal porphyrin-like plane, which in the optimized structure results to be arranged in a more planar fashion with respect to the crystallographic one (red in Figure 2). Accordingly, in Figure 2 all the rings seem to be not superimposable. However, looking at the reported structural parameters for each structure, the differences between them are much less pronounced, with the exception of those parameters that account for the planarity of the four N-rings, such as ϕ_1_ and ϕ_2_. Also the orientation of the *meso*-Ar rings in the optimized geometry is slightly different from those that came out from the crystallographic characterization (see ϕ_4_ values). In any case, it is noteworthy that the computed structure is obtained optimizing in gas-phase, unencumbered by disturbing factors, which necessarily does not take into account constrains imposed by the crystallographic characterization.

### 2.2. PDT-Related Properties

**Figure 3:**
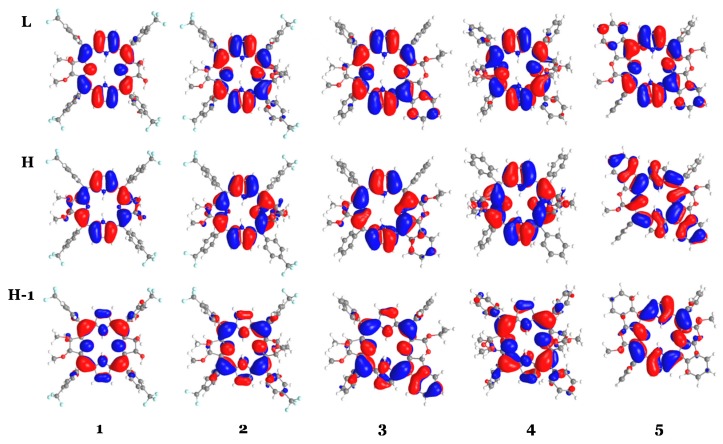
Graphical representation of the HOMO-1, HOMO and LUMO for compounds **1**–**5** computed at B3LYP/6-31G* level of theory.

The computed vertical excitation energies for the two separated peaks within the Q band, suitable for PDT of deeper tumor tissues, are reported in Table 1 together with the available experimental λ_max_ values. All the employed exchange-correlation functionals correctly predict the nature of the Q_x_ and Q_y_ bands, which are originated from HOMO-1 and HOMO to LUMO transitions, respectively (see Table 1 and Figure 3).

The range separated hybrid functional ωB97XD is able to reproduce with good accuracy the experimental Q_y_ wavelength, showing an average error of only 10 nm against the experimental value. The ωB97XD good performances have been also reported in previous study on a series of molecules with extended conjugation [45]. B3LYP and M06 functionals instead, predict a blue-shifted band affected by an average error of 82 and 56 nm with respect to the experimental counterparts, respectively. On the contrary, the Q_x_ band is computed at higher wavelengths by all the XC functionals tested, with average errors of 19, 28 and 35 nm against the experimental value [36] for B3LYP, ωB97XD and M06 functionals, respectively.

In agreement with experimental evidences, a red shift of approximately 50 nm is found going from the dihydroxydimethoxybacteriochlorin **1** to the morpholinobacteriochlorin **2**, in which the insertion of an oxygen atom between the two *sp*^3^ hybridized pirrolidone β carbons, leads to a more distorted conformation (see Figure 1). The red-shift effect associated with the loss of planarity in porphyrin-like systems, has been previously evidenced [36]. Compared to the parent bacteriochlorin **2**, the β-*o*-phenyl-morpholinobacteriochlorin **3** shows a blue-shifted Q_y_ band. Indeed, despite the extended π conjugation of the chromophore generated by the fusion of the *meso*-phenyl group to the morpholine moiety should produce a shift toward higher wavelengths, the quite planar conformation generated by the β-*o*-phenyl linkage predominates on its optical properties producing an overall blue-shift of the band. The bismorpholinobacteriochlorin (**4**) contains two morpholino groups in the bacteriochlorin ring, whose inherent twist produces a significant skeleton distortion accompanied by a further red-shift of the Q_y_ band.

Finally, compound **5**, in which two β-*o*-phenyl groups are linked to the morpholino ones, shows a Q_y_ transition that, despite the presence of a greater number of π electrons, is blue-shifted compared to **4**. Again, conformational effects dominate over electronic ones. The increased rigidity of the skeleton produced by the β-to-*o*-phenyl linkages, analogously to what observed for molecule **3**, causes a slight planarization of the molecule and a consequent hypsochromic shift of the band. Similar modulation of the spectra dominated by conformational changes in expanded macrocycles has been recently reported [44].

The absorption band at the highest wavelength found for compound **4**, is in agreement with the lowest HOMO-LUMO gap computed for the same molecule, equal to 2.09 eV.

A photosensitizer suitable to be used in PDT must have also an energy gap between singlet ground and low-lying triplet excited states (ΔE_S-T_) greater than the energy needed to generate the cytotoxic singlet oxygen species. At the same level of theory used in this study, B3LYP/6-31G*, the energy required to excite the triplet molecular oxygen has been computed to be 0.91 eV, in good agreement with the experimental value (0.98 eV).

**Figure 4:**
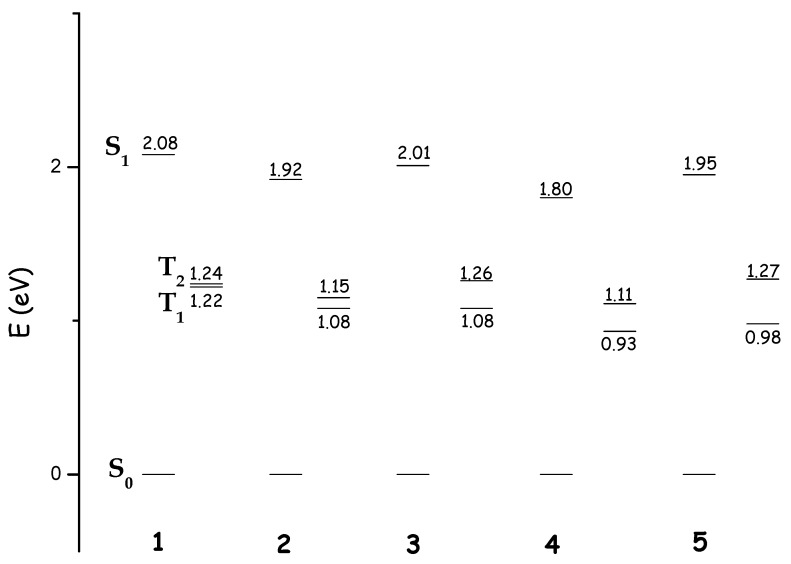
Lowest vertical singlet and triplets excitation energies (eV) of compounds **1**–**5** computed in dichloromethane at TD-B3LYP/cc-pVDZ level of theory.

As can be seen in Figure 4, all the considered compounds exhibit a vertical ΔE_S-T_ for the low lying excited triplet state greater than 0.91 eV and, in principle, are all able to produce the ^1^O_2_ (^1^Δ_g_). However, the probability that singlet oxygen is produced depends on the effectiveness of the non-radiative transition from singlet excited state S_1_ to the low lying triplet ones.

As the intersystem spin crossing efficiency essentially depends on the amplitude of the Spin-Orbit matrix elements for the S_1_ → T_j_ radiationless transitions, these quantities have been determined by using the atomic mean field approximation for all the studied molecules and collected in Table 2.

As shown in Figure 4, upon photoexcitation, there are two possible ISC channels for the spectroscopic state S_1_ in all the investigated bacteriochlorins, S_1_ → T_1_ and S_1_ → T_2_. Because of the comparable energetic gap between S_1_ and T_j_ (j = 1, 2) especially for **1** and **2** bacteriochlorins, conceivably both the radiationless could ably contribute to the intersystem spin crossing efficiency.

Samankumara *et al.* determined the intersystem crossing quantum yield for some of the compounds investigated here, showing how the introduction of the morpholine moiety (**2**) and the establishment of the β-*o*-phenyl linkage (**3**) entail an increase and decrease of the ISC quantum yield, respectively [36]. Accordingly, looking at data reported in Table 2, with respect to the simple bacteriochlorin **1**, the computed spin-orbit matrix elements of morpholinobacteriochlorin **2** for both the S_1_ → T_j_ transitions are significantly greater than the former one (3.3 *vs.* 1.3 and 3.4 *vs.* 0.8, respectively), as well as those of molecules **3** while **5** remains very similar to **1**.

Similarly, the computed SOCs for bismorpholinobacteriochlorin **4** result to be greater than those found for **1**, because of its non-planar and flexible conformation, although they are slightly smaller than those computed for monomorpholinobacteriochlorin **2**.

Considering that the SOC value for S_1_ → T_1_ transition of Foscan©, currently approved for PDT [47] is computed to be 0.25 cm^−1^ [9], keeping in mind also the other requirements fulfilled by the investigated bacteriochlorins, all of them can undergo efficient intersystem crossing with consequent production of cytotoxic singlet molecular oxygen. Besides, it is worthy of note that according to the El Sayed rules, as the nature of the molecular orbitals involved in these transitions (are all π-π*) remain the same, the computed SOCs are rather small, although SOC values between 0.2 and 5.0 cm^−1^ are considered large enough to induce ISC on a nanosecond time scale [48].

Hence, computational results confirm the trend of ISC quantum yield experimentally determined, suggesting that the introduction of the morpholine moiety represents the best expansion of bacteriochlorin core to enhance the ability to produce singlet oxygen species.

## 3. Computational Methods

All the calculations have been done by using Gaussian 09 code [49]. The structures have been optimized without any constrains by using B3LYP exchange and correlation functional [50,51] coupled with 6-31G* basis set for all the atoms.

Absorption spectra have been obtained as vertical electronic excitations from the minima of the ground-state structures by using time-dependent density functional response theory (TD-DFT) [52].

Two hybrid exchange and correlation functionals, B3LYP and M06 [53], and a range-separated hybrid functional, ωB97XD [54], in conjunction with the 6-31+G* basis set have been employed.

The solvent environment, dichloromethane, has been simulated by means of the integral equation formalism polarizable continuum model (IEFPCM) [55,56], which corresponds to a linear response in non-equilibrium solvation, with a dielectric constant of 8.93.

Spin-orbit matrix elements have been computed using the quadratic-response TD-DFT approach [57,58], as implemented in the Dalton code [59], at their ground state optimized geometries in the framework of the atomic-mean field approximation [60]. For this purpose, B3LYP coupled with the cc-pVDZ basis set for all the atoms has been used. The spin-orbit couplings (SOCs) have been defined according to the following formula:

$$SOC_{ij}=\sqrt{\underset{n}{\sum }{\left|\psi_{S_{i}}\left|{\hat{H}}_{SO}\right|\psi_{T_{j,n}}\right|}^{2}};\;n=x,y,z$$

where ${\hat{H}}_{SO}$ is the spin-orbit Hamiltonian.

The triplet-singlet energy gap of molecular oxygen O_2_ has been evaluated at B3LYP/6-31+G* level of theory. The $S^{2}$ values have been checked to evaluate whether the energy of the two states could be affected by spin contamination. While for the triplet state a $S^{2}$ value very close to 2.0 has been found, the unrestricted calculation of the singlet energy gave a ^1^Δ_g_ state too much stable due to the contamination of the singlet wave function with that of the triplet state ($S^{2}≅1.0$). Adopting the method proposed by Ovchinnikov and Labanowski to correct the mixed spin energies and removing the foreign spin components [61], the singlet state corrected energy and a triplet-singlet energy gap of 0.91 eV were obtained, in very good agreement with the experimental value of 0.98 eV.

## 4. Conclusions

In this work the time-dependent density functional response theory has been employed to compute, for a series of extended bacteriochlorins, the most important photophysical properties (excitation energies, singlet-triplet energy gap and spin-orbit matrix elements) that can be useful to propose a photosensitizer as PDT agent. On the basis of our results, the following conclusions can be outlined:

1. All the investigated bacteriochlorins show a maximum absorption wavelength that falls in the therapeutic window (600–800 nm);
2. The Q_y_ wavelength is better reproduced by ωB97XD exchange-correlation functional, although the Q_x_ band transition energy is computed with good accuracy by all the employed XC functionals;
3. The most red-shifted transitions have been displayed by systems in which the extension of the bacteriochlorins core entails the largest degree of strain (**2**, **4**);
4. All the considered systems show singlet-triplet energy gaps great enough to excite the molecular oxygen from its ^3^Σ_g_ˉ ground state to the singlet ^1^Δ_g_ excited one;
5. The SOCs computed for all bacteriochlorins result to be higher than that computed for Foscan©, which is currently used in the medical PDT protocols.

Considering the three photophysical properties investigated here, among the five studied systems, the expanded bacteriochlorins containing morpholino group (**2** and **4**) can be proposed as the better photosensitizers for type II photoreactions. We hope that our work can stimulate further experimental works on these interesting molecules.

## Figures and Tables

**Table 1:** Main vertical singlet electronic energies ΔE (eV, nm), oscillator strengths, *f*, and main configuration for **1**–**5** compounds in dichloromethane solvent computed by employing the 6-31+G* basis set at ωB97XD, M06 and B3LYP level of theory on the B3LYP/6-31G* optimized geometries. Experimental values in nm are taken from reference [36].

Com.	Band	MO Contribution	B3LYP		ωB97XD		M06		
			ΔE	*f*	ΔE	*f*	ΔE	*f*	*exp*
**1**									
	Q_y_	H → L (91%)	2.00, 618	0.360	1.77, 702	0.367	1.92, 644	0.367	707
	Q_x_	H-1 → L (81.9%)	2.32, 534	0.237	2.26, 548	0.246	2.26, 549	0.212	524
**2**									
	Q_y_	H → L (92%)	1.86, 667	0.292	1.64, 754	0.299	1.78, 695	0.299	745
	Q_x_	H-1 → L (80%)	2.21, 560	0.218	2.15, 575	0.232	2.15, 576	0.198	544
**3**									
	Q_y_	H → L (88%)	1.96, 634	0.293	1.75, 705	0.300	1.89, 657	0.298	715
	Q_x_	H-1 → L (87%)	2.12, 585	0.362	2.08, 596	0.365	2.07, 600	0.338	563
**4**									
	Q_y_	H → L (92%)	1.75, 710	0.245	1.55, 798	0.245	1.67, 741	0.249	790
	Q_x_	H-1 → L (81%)	2.09, 591	0.221	2.04, 608	0.231	2.04, 608	0.206	562
**5**									
	Q_y_	H → L (74%)	1.89, 656	0.367	1.73, 716	0.284	1.83, 677	0.349	735
	Q_x_	H-1 → L (68%)	2.01, 618	0.342	1.95, 635	0.426	1.95, 634	0.339	598

**Table 2:** Spin-orbit coupling Cartesian components and correspondent spin-orbit couplings (SOC) (cm^−1^) between low-lying singlet and triplet excited states calculated at B3LYP/cc-pVDZ//B3LYP/6-31G* level of theory.

	1	2	3	4	5
$\langle \Psi_{S_{1}}|{\hat{H}}_{so}|\Psi_{T_{1}}\rangle$	4.6 × 10^−2^ (x)	2.5 × 10^−1^	4.4 × 10^−1^	2.2 × 10^−3^	0
	1.3 (y)	2.9	5.4 × 10^−1^	6.6 × 10^−3^	0
	4.2 × 10^−2^ (z)	1.4	9.2 × 10^−1^	2.8	1.4
SOC	1.3	3.3	1.2	2.8	1.4
$\langle \Psi_{S_{1}}|{\hat{H}}_{so}|\Psi_{T_{2}}\rangle$	1.5 × 10^−1^ (x)	6.4 × 10^−1^	3.9 × 10^−1^	2.2 × 10^−1^	0
	7.7 × 10^−1^ (y)	3.0	3.1 × 10^−1^	0.0	0
	1.3 × 10^−2^ (z)	1.5	2.6 × 10^−1^	2.5	6.7 × 10^−1^
SOC	0.8	3.4	0.4	2.6	0.7
